# Genetic insights into autism spectrum disorder with intellectual disability: a regional population-based study from Northwest China

**DOI:** 10.3389/fped.2026.1829150

**Published:** 2026-06-25

**Authors:** Yanrui Dai, Rui Yao, Tianju Du, Xiaochen Wang, Nan Dou, Feixuan Zhang, Li Liu

**Affiliations:** 1 Department of Pediatric Rehabilitation, The First People’s Hospital of Yinchuan, Yinchuan, China; 2 Second College of Clinical Medicine, Ningxia Medical University, Yinchuan, China

**Keywords:** autism spectrum disorder, gene, genetic etiology, intellectual disability, whole-exome sequencing

## Abstract

**Objective:**

To investigate the genetic etiology of autism spectrum disorder with Intellectual Disability in the Northwest China.

**Methods and results:**

Whole-exome sequencing was conducted on 125 children admitted to Yinchuan First People's Hospital. Variants and associated pathogenic mechanisms were analyzed, as well as correlations between genotypes and clinical phenotypes. Forty-five positive cases were identified from 125 cases, yielding a positive detection rate of 36.0%, with a male-to-female ratio of 2:1. These included 8 cases with copy number variations and 37 cases of single-nucleotide variations/insertions and deletions. Chi-squared tests and False Discovery Rate adjust showed significant differences in gross motor developmental delay and abnormal electroencephalograms between the positive group and part of the negative group (*q* < 0.05). Further, there was a significant difference in autism spectrum disorder severity between the positive and negative groups (*χ*^2^ = 10.20, *P* < 0.01).

**Conclusion:**

This study identified several pathogenic genes that have been rarely reported in the context of autism spectrum disorder. Patients with these genetic variants may present with more complex and severe clinical phenotypes.

## 1 Introduction

Autism spectrum disorder (ASD) is a neurodevelopmental disorder (NDD) characterized by core features, such as impaired social interactions, communication difficulties, restricted and repetitive behaviors (RRBs), and narrow interests (1). Autism cases are often accompanied by other NDDs rather than occurring in isolation, such as intellectual disability (ID). ASD diagnoses are steadily increasing, with recent studies in the USA indicating a childhood ASD prevalence of 2.76%, with a male-to-female ratio of 3.8:1, associated with severe functional impairments and social deficits in affected children (2).

To date, the etiology of ASD remains complex and undefined, with genetic factors recognized as one of the primary causes. Recent technological advances, including developments such as first-generation sequencing, whole-exome sequencing (WES), whole-genome sequencing (WGS), and chromosomal microarray analysis (CMA), have led to reports of chromosomal variations, copy number variants (CNVs), small insertions/deletions (indels), single-nucleotide variants (SNVs), and epigenetic-related variations associated with ASD (3). Gyawali et al (4). reported an ASD heritability ranging from 74% (with a prevalence rate of 1%) to 93% (with the prevalence rate increasing to 5%). In addition to the recognition of genetic factors as the main pathogenic drivers, mechanisms such as shared pathways among related genes are increasingly recognized as critical to the development and progression of ASD. Therefore, investigation of the genetic etiology of ASD is important for improving the early recognition, diagnosis, and treatment of the disorder.

## 2 Material and methods

### 2.1 Subjects

A total of 125 children with ASD and ID, aged 1 to 10 years, who were treated in the Department of Child Rehabilitation at the Yinchuan First People's Hospital between 2021 and 2024 were enrolled. Of these, 92 were male, and 33 were female, resulting in a sex ratio of 2.8:1. Clinical information, imaging data, and the results of developmental and ASD assessment scales, including the autism behavior checklist (ABC) and the childhood autism rating scale (CARS), were collected. The ABC is used for ASD screening, while CARS is a diagnostic scale (The study utilized the Chinese version of ABC, originally developed by Krug et al. (1980) and validated in Chinese populations, and CARS, based on the standard version by Schopler et al. (1980) with established reliability in clinical autism assessments). In this study, a preliminary screening was conducted using the ABC, followed by an ASD diagnosis using a combination of the DSM-5 and CARS, with a final classification of disease severity according to the scoring criteria of the CARS; ID diagnosis was performed using a combination of DSM-5 and the Wechsler Scales (WISC-IV and WPPSI-IV). Inclusion criteria: 1) Meeting the diagnostic criteria for ASD with ID; 2) Informed consent from family members for family WES. Exclusion criteria: 1) A clear history of craniocerebral trauma or organic neurological diseases such as encephalitis; 2) Incomplete clinical data. This study was approved by the Ethics Committee of our hospital (KY-2021-024), and informed consent from the family members was obtained.

### 2.2 Methods

#### 2.2.1 Case data

Whole-exome sequencing was performed on 125 children who met the inclusion criteria. Pathogenic (P) and Likely Pathogenic (LP) variants were recognized as positive results, and clinical data of positive cases and some negative cases (only those with complete data were included) were collected. According to the CARS criteria, ASD was classified as mild-moderate (30‒35 points) and severe (≥36 points).

#### 2.2.2 Genomic DNA extraction

Two milliliters of peripheral venous blood were collected from the children and their parents into EDTA anticoagulant tubes and sent to the Molecular Medicine Research Center(Molecular Medicine Center, Children's Hospital of Fudan University) for testing. Genomic DNA was extracted from the peripheral blood and analyzed by WES (Sequencing was performed using an Illumina next-generation sequencer with an average depth of 100  ×  ).

An integrated diagnostic platform combining molecular annotations and biological, genetic, and clinical feature analyses, along with information on disease-causing mutations and normal population genomes from databases. Variants are annotated using an automated monitoring system specifically designed for second-generation sequencing experiments and associated analytical workflows(Reference genome: GRCh37/hg19). Additionally, per-base sequencing depth and variant predictions are derived from all available genomic sequencing data. The annotation process is performed using Alamut Batch software, with reference to the ClinVar, HGMD and OMIM databases, which contain curated information on known or suspected pathogenic variants, and algorithms for the analysis of genetic data, was used to classify and filter out millions of genetic variants (including variants in non-coding regions within 150 bp upstream and downstream of the coding region and the UTR region).

Variants were classified based on American College of Medical Genetics and Genomics (ACMG standards, combining the latest online genetic research on ASD and the expert interpretations in genetics, and potential structural variants were annotated and analyzed. The corresponding sequences in the patients’ parents were analyzed using Sanger sequencing and CNV-seq.

#### 2.2.3 Data collation and analysis

Based on the results of the genetic testing, the samples were divided into positive and negative groups. The positive group was further subdivided into CNV and SNV/Indel groups according to genomic structural differences. The genetic distribution characteristics of children with autism and intellectual disability in this region were reviewed and analyzed.

#### 2.2.4 Statistical methods

Data were analyzed using SPSS 26.0 software. Chi-square tests (*n* ≥ 40 and T ≥ 5) were used to compare differences in the primary clinical data and ASD severity between the positive and negative groups. The Benjamini-Hochberg method was employed to control the false discovery rate (FDR). Multiple test corrections were applied to the comparison results of the six clinical phenotypes. The adjusted q-values were calculated, and a difference was considered statistically significant if q < 0.05.

## 3 Results

### 3.1 Genetic sequencing and clinical data of the positive group

A total of 45 positive cases were identified, including 8 cases with CNVs and 37 cases with SNV/Indels, with an overall positive detection rate of 36.0%. The male-to-female ratio was 30:15 (2:1). The age distribution of the confirmed pathogenic genes was as follows: 1–3 years (21 cases), 4–6 years (19 cases), and 7–9 years (5 cases).

### 3.2 Results of the CNV group

Eight positive CNV cases were detected, with a male-to-female ratio of 7:1 and a detection rate of 6.4%. Among these, 7 deletions and 2 duplications were identified, with one child having 2 CNVs, namely, a deletion variant in *SHANK* and a 0.16 Mb duplication variant. These CNVs involved two cases each of 15q11.2q13.1 and 22q13.31q13.33, and 1 case each of the remaining regions (Table 1).

**Table 1:** Genetic results of the positive CNV group.

Patient	Sex	Age (y)	Chromosomal region	Type	Segment size (Mb)	Origin	Key genes	Related disorders	Severity
1	M	2	1p36.33p36.31	del	5.23	*de novo*	GNB1	1p36 Microdeletion Syndrome	Severe
2	M	2	22q13.33	del	0.18	*de novo*	SHANK3	Phelan-McDermid Syndrome	Severe
3	M	2	15q11.2q13.1	del	7.82	*de novo*	GABRB3, GABRB5	Early-onset Infantile Epileptic Encephalopathy	Mild-Moderate
4	M	3	chr17p11.2	dup	0.16	*de novo*	—	—	Mild-Moderate
			22q13.31-q13.33	del	3.18	*de novo*	SHANK3	22q13.3 Deletion Syndrome	Mild-Moderate
5	F	2	16p13.11	del	1.55	Maternal	MYH11, ABCC6	16p13.11 Microdeletion Syndrome	Mild-Moderate
6	M	5	7q11.23	dup	0.85	*de novo*	YWHAG, POR	—	Mild-Moderate
7	M	5	14q32.2	del	3.03	*de novo*	BCL11B, VRK1	Intellectual Developmental Disorder	Severe
8	M	2	15q11.2-q13.2	del	6.80	*de novo*	GABRA5, UBE3A	Angelman Syndrome	Mild-Moderate

The results of all eight children were verified by Sanger sequencing and CNV-seq. This showed that seven cases were *de novo* variants and one case was inherited from the mother; the *de novo* variants thus accounted for 87.5%, a relatively high proportion. Analysis of clinical data showed that four children had abnormal brain MRI findings, including three cases of Dandy-Walker malformation and one case of tuberous sclerosis complex.

### 3.3 Results of the SNV/indel group

A total of 37 positive cases with SNV/Indel variants associated with ASD were identified by WES, with a detection rate of 29.6%. These involved 37 mutated genes, including 34 SNVs and 3 indels. The genes included *ACSL4, SHANK3, MECP2, HIVEP2, GDI1, DNMT3A, SYNGAP1, SCN2A, TSC2, TBR1, SIN3A,* and *TCF4*. Among them, variants in *SYNGAP1, SCN2A, TSC2, GDI1*, and *THOC2* were each detected in two cases, and variants in the remaining genes were observed in one case (Table 2).

**Table 2:** Genetic results of the positive SNV groups.

No.	Sex	Age (y)	Gene	Variant position	Inheritance pattern	Disorders	Severity
1	M	5	*ACSL4*	chrX:c.1202G > T,p.G401 V,MS	XL/Het/M	X-linked mental retardation 63	Severe
2	W	4	*HIVEP2*	chr6:c.3556C > T,p.Q1186X,MS	AD/Het/DN	Intellectual developmental disorder, autosomal dominant 43	Mild-Moderate
3	M	3	*GDI1*	chrX:c.1244A > G,p.N415S,MS	XL/Hem/M	Intellectual developmental disorder, X-linked 41	Mild-Moderate
4	M	3	*SIK1*	chr21:c.2185G > C,p.D729H,MS	AD/Het/M	Developmental and epileptic encephalopathy 30	Mild-Moderate
5	M	3	*SYNGAP1*	chr6:c.3163G > A,p.G1055R,MS	AD/Het/P	Intellectual developmental disorder, autosomal dominant 5	Mild-Moderate
6	M	3	*CNOT3*	chr19:c.970C > T,p.P324S,MS	AD/Het/M	Intellectual developmental disorder with speech delay, autism	Mild-Moderate
7	M	3	*TRIO*	chr5:c.9016C > T,p.R3006C,MS	AD/Het/DN	Intellectual developmental disorder, autosomal dominant 44	Mild-Moderate
8	W	6	*SYNGAP1*	chr6:c.3557C > A,p.Ser1186*,NS	AD/Het/DN	Intellectual developmental disorder, autosomal dominant 5	Severe
9	W	3	*KDM6B*	chr17:c.949G > A,p.A317 T,MS	AD/Het/DN	Stolerman neurodevelopmental syndrome	Severe
10	W	6	*HERC2*	chr15:c.8329A > G,p.M2777 V,MS	AR/Het/M	Intellectual developmental disorder, autosomal recessive 38	Mild-Moderate
				chr15:c.8558-3T > C,SV	AR/Het/P		
11	W	5	*MAP1B*	chr5:c.5819A > G,p.Y1940C,MS	AD/Het/P	Periventricular nodular heterotopia 9	Mild-Moderate
12	M	2	*THOC2*	chrX:c.1372T > C,p.S458P,MS	XL/Hem/M	Intellectual Developmental Disorder, X-linked 12	Mild-Moderate
13	M	3	*USP9X*	chrX:c.5974C > T,p.R1992W,MS	XL/Hem/M	Intellectual developmental disorder, X-linked 99	Severe
14	M	8	*PDZD8*	chr10:c.851C > G,p.T284S,MS	AR/Hom/PM	Intellectual developmental disorder with autism	Severe
15	M	5	*GDI1*	chrX:c.280A > G,p.T94A,MS	XL/Hem/M	Intellectual developmental disorder, X-linked 41	Mild-Moderate
16	W	8	*DNMT3A*	chr2:c.988T > C,p.W330R,MS	AD/Het/DN	Tatton-Brown-Rahman syndrome	Mild-Moderate
17	W	6	*SHANK3*	chr22:c.4429:G > A,p.G1477R,MS	AD/Het/M	Phelan-McDermid syndrome	Mild-Moderate
18	M	8	*NIPBL*	chr5:c.6944:A > G,p.H2315R,MS	AD/Het/DN	Cornelia de Lange syndrome 1	Severe
19	W	3	*SCN2A*	chr2:c.305G > A,p.R102Q,MS	AD/Het/DN	Developmental and epileptic encephalopathy 11	Severe
20	W	3	*MECP2*	chrX:c.441A > C,p.K147 > N,MS	XL/Het/DN	Rett syndrome,Autism, X-linked 3	Severe
21	W	3	*AGO2*	chr8:c.879-13C > T,SV	AD/Het/P	Lessel-Kreienkamp syndrome	Mild-Moderate
22	M	5	*CAMTA1*	chr1:c.2600G > A,p.S867N,MS	AD/Het/M	Cerebellar dysfunction with behavioral abnormalities	Mild-Moderate
23	M	3	*FOXP1*	chr3:c.152T > C,p.L51P,MS	AD/Het/P	Intellectual developmental disorder with autistic features	Mild-Moderate
24	M	7	*NF1*	chr17:c.4084C > T,p.R1362*,NS	AD/Het/DN	Neurofibromatosis, type 1	Severe
25	W	1	*TSC1*	chr9:c.2356C > T,P.R786*,NS	AD/Het/M	Tuberous sclerosis-1	Severe
26	M	3	*SUPT16H*	chr14:c.2693A > T,P.Q898L,MS	AD/Het/DN	Neurodevelopmental disorder with dysmorphic facies	Severe
27	M	5	*TSC2*	chr16:c.2513del,p.S838Tfs*56	AD/Het/DN	Tuberous Sclerosis-2	Mild-Moderate
28	M	9	*PAH*	chr12:c.1162G > A,p.V388M,MS	AD/Het/DN	Phenylketonuria	Severe
				chr12:c.1200-8G > A,MS	AD/Het/DN		
29	M	6	*CREBBP*	chr16:c.3016A > G,p.T1006A,MS	AD/Het/P	Menke-Hennekam syndrome 1	Mild-Moderate
30	W	3	*SCN2A*	chr2:c.3G > A,p.M1I,MS	AD/Het/DN	Developmental and epileptic encephalopathy 11	Severe
31	M	4	*THOC2*	Xq25:c.4519 + 14T > C,SV	XL/Hem/M	Intellectual developmental disorder, X-linked syndromic,type 12	Severe
32	M	4	*TSC2*	16p13.3:c.1716G > T,MS	AD/Het/DN	Tuberous Sclerosis-2	Severe
33	M	5	*SCN1A*	chr2:c.2214G > A,p.W738*,NS	AD/Het/DN	Dravet syndrome	Mild-Moderate
34	W	1	*TBR1*	chr2:c.1092-1093del,p.E365Vfs*29	AD/Het/DN	Intellectual developmental disorder with autism	Mild-Moderate
35	W	2	*SIN3A*	chr15:c.2709_2712del,p.C903*,NS	AD/Het/DN	Witteveen-Kolk syndrome	Mild-Moderate
36	M	6	*TCF4*	chr18:c.955C > T:p.Gln319*,MS	AD/Het/DN	Pitt-Hopkins syndrome	Severe
37	M	5	*BRAF*	chr7:c.1787G > T,p.G596 V,MS	AD/Het/DN	LEOPARD syndrome 3; Cardiofaciocutaneous syndrome	Severe

ACMG has provided two sets of criteria: one for classification of P or LP variants, and one for classification of Benign or Likely Benign variants, each pathogenic criterion is weighted as very strong (PVS1), strong (PS1–4); moderate (PM1–6), or supporting (PP1–5). The pathogenic variants listed in Table 2 were all strictly screened according to the aforementioned standards of ACMG. For example, as per ACMG evidence: pathogenic (PVS1, PM2, PP3).

FS, Frameshift mutation; MS, Missense mutation; SV, Splice variant; NS, Nonsense mutation; AD, Autosomal dominant inheritance; AR, Autosomal recessive inheritance; XL, X-linked inheritance; Het, Heterozygous; Hom, Homozygous; Hem, Hemizygous; P, Paternal; M, Maternal; DN, *de novo*.

Classification according to Mendelian inheritance patterns identified 27 cases of autosomal dominant (AD) inheritance, 7 cases of X-linked (XL) inheritance, and 3 cases of autosomal recessive (AR) inheritance, which included 1 homozygous variant and 2 compound heterozygous variants. AD inheritance accounted for the highest proportion (72.97%). Sanger sequencing verification of the mutant sites in the probands was performed on the parents of the 37 children, revealing 19 *de novo* gene variants, 11 variants inherited from the mother, 5 from the father, and 2 from both parents. The *de novo* variants accounted for the highest proportion (51.35%), followed by maternal inheritance (29.7%), paternal inheritance (13.5%), and biparental inheritance (5.4%) (Table 2).

Among the 37 mutated genes identified in this study, 39 variant sites were observed (two cases with compound heterozygous variants), including 27 missense mutations (MS), 6 nonsense mutations (NS), 4 splice variants (SV), and 2 frameshift mutations (FS), accounting for 69.23%, 15.38%, 10.26%, and 5.13% respectively. These variants involved multiple chromosomes and mutation sites, and included different types of variants. Some children exhibited multiple clinical phenotypes apart from the core symptoms of ASD, showing comorbidity with other NDDs, such as ADHD and epilepsy, reflecting the genetic and clinical heterogeneity of ASD (Table 2).

### 3.4 Correlation between genotype and clinical phenotype

Chi-square tests and FDR adjust were used to compare clinical phenotypes between the positive and negative groups. The results showed statistically significant differences in gross motor developmental delay and abnormal EEG findings (*q* < 0.05), while no significant differences were observed in other phenotypes (Table 3).

**Table 3:** Comparison of clinical phenotypes between the positive and negative groups (n%).

Clinical phenotype	Positive group (*n* = 45)	Negative group (*n* = 80)	*χ*^2^ value	*P* value	Corrected q value
Gross motor development delay	26 (57.78%)	16 (20.00%)	18.423	＜0.001**	0.007
Abnormal EEG	16 (35.55%)	12 (15.00%)	7.002	0.008**	0.028
Abnormal brain MRI	15 (33.33%)	13 (16.25%)	4.836	0.028*	0.065
Hearing impairment	10 (22.22%)	10 (12.50%)	2.026	0.155	0.217
Epilepsy	8 (17.78%)	6 (7.50%)	3.058	0.080	0.140
ADHD	7 (15.55%)	7 (8.75%)	1.341	0.247	0.247

* *P* < 0.05

** *P* < 0.01.

Chi-square tests were used to compare the severity of ASD between the positive and negative groups, utilizing fourfold table data. The results showed *χ*^2^ = 10.20, *P* < 0.01, indicating a significant difference in ASD severity between the positive and negative groups (Table 4).

**Table 4:** Comparison of ASD severity between the positive and negative groups (n%).

ASD severity	Mild/moderate	Severe	Total
Positive group	25 (55.55)	20 (44.45)	45
Negative group	52 (82.54)	11 (17.46)	63
Total	77	31	108

*P*＜0.01 (*χ2* = 10.20).

### 3.5 Genetic pathogenesis

Thirty-seven ASD-related genes were identified. Using the STRING database (https://cn.string-db.org/) and Cytoscape software to compile a “gene interaction” network, it was found that multiple core genes (including *MECP2, SHANK3,* and *CREBBP*) play crucial roles in ASD pathogenesis, contributing collectively to its development.

## 4 Discussion

Autism spectrum disorder is a highly complex neurodevelopmental disorder characterized by significant heterogeneity in genetic etiology, clinical manifestations, and penetrance. Here, WES was performed on children meeting the diagnostic criteria of ASD, resulting in an overall 36% rate of genetic variant detection, including a 29.6% rate of SNV/Indel and 6.4% rate of CNV identification. This detection rate is slightly higher than that described by Ishay et al., likely due to the subjects about ASD comorbid with ID, which may have increased the diagnostic yield of WES in the present study (5, 6).

### 4.1 CNV variants

Five ASD-related CNVs were identified, specifically at 1p36.33-p36.31, 14q32.2, and 16p13.11, as well as duplications at 7q11.23 and 17p11.2. These variants have not been known in previous studies on ASD genetics, possibly due to regional specificities, the inclusion of patients with comorbid NDDs, and differences in sample size, disease severity, or technical factors such as the current analytical capabilities of genetic tests and sequencing coverage. Eight positive CNVs were found, with a male-to-female ratio of 7:1, indicating a significantly higher rate of CNV detection in male patients. A previous epidemiological study of ASD reported a male-to-female ratio of 3.8:1, with males showing an 11% higher proportion of congenital genetic risk factors (2). This may be explained by the “female protective effect,” whereby female patients typically require a higher genetic burden for symptom manifestation. Although they may carry the same variants as boys, they often remain asymptomatic (7), resulting in a relatively higher detection rate of pathogenic CNVs in females.

### 4.2 SNV/indel variants

In 2017, Vorstman et al. (8) reported that autosomal dominant (AD) inheritance accounts for 20% of ASD cases based on Mendelian genetic principles. However, the proportion of AD variants identified in the present study was significantly higher than observed previously, with *de novo* mutations comprising 63.0% of AD cases. This discrepancy may be attributed to the increased use of WES in disease detection, leading to improved identification of *de novo* variants. *De novo* mutations with AD inheritance play a significant role in ASD, particularly in sporadic cases. Consistent with this, most of the children in the present study had no family history of ASD, indicating sporadic occurrence. Previous studies by Kong et al. and Jónsson et al. (9, 10) found that paternal age is a major driver of *de novo* mutations, with approximately 97% of such variants originating from the father. This is likely due to the increased number of cell divisions in male germ cells with advancing paternal age, elevating the likelihood of DNA replication errors. With the implementation of China's three-child policy, paternal age at conception has increased, potentially raising the risk of ASD in offspring.

This study found that autosomal recessive (AR) variants accounted for 8.1% of cases, including one patient (Case 14) with homozygous variants who was born to consanguineous parents (a maternal uncle and a paternal aunt). The patient presented with severe ASD. A study by Lim et al. (11) in 2013 highlighted the importance of rare homozygous mutations in ASD, especially in consanguineous families. The study samples were collected from remote areas inhabited by ethnic minorities, where cultural and traditional practices may lead to higher rates of consanguineous marriage compared to developed regions of China, thereby increasing the incidence of homozygous variants in ASD.

Moreover, the X-linked genetic disorders accounted for 18.9% of cases in the present study, a higher proportion than previously reported. This may be associated with factors such as higher fertility rates in the Ningxia region (increasing the penetrance of X-linked disorders, particularly in males), consanguineous marriage, and higher frequencies of X-linked variants in ethnic minority populations (8).

**Figure 1:**
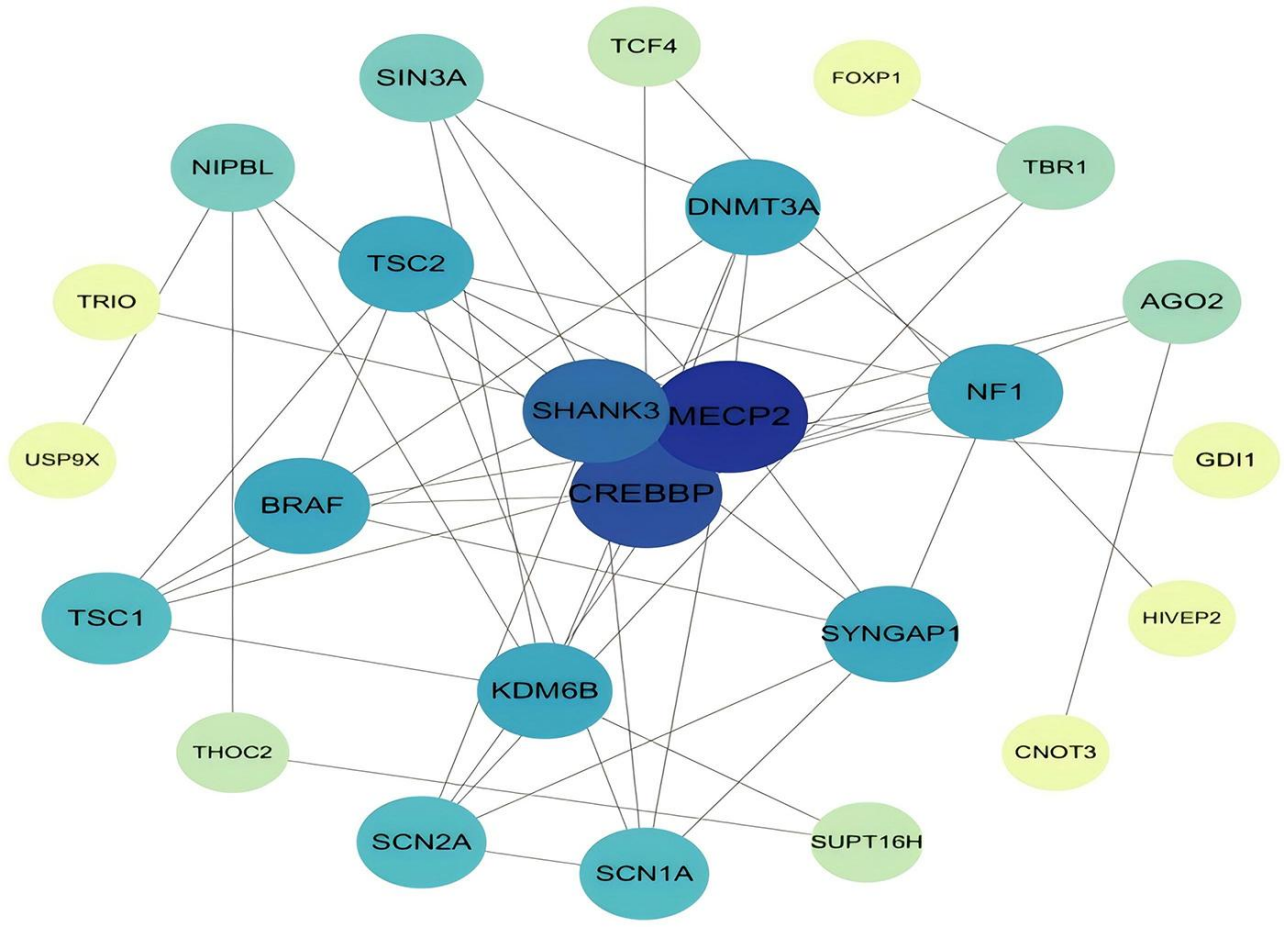
Gene interaction network.

The present findings suggest that ASD-related genes influence each other through genetic interactions, co-expression, and shared protein domains. For example, core genes including *MECP2, CREBBP, SHANK3*, and *TSC2* are interconnected, indicating shared functional pathways and close associations in biological functions or signaling pathways (Figure 1). Previous studies by Jung et al. and Karimi et al. (12, 13) confirmed that *MECP2, SHANK3*, and *CREBBP* are pathogenic or high-risk genes for ASD. SHANK3, a core scaffolding protein in the postsynaptic density (PSD), links neurotransmitter receptors, ion channels, and other membrane proteins, playing a critical role in synaptogenesis, dendritic spine maturation, and the maintenance of synaptic plasticity, thereby influencing neuronal morphology and function (14). Point mutations or CNVs in *SHANK3* are associated with a variety of NDDs, such as ASD, intellectual disability, and schizophrenia, particularly ASD.

### 4.3 Syndromic autism spectrum disorder

According to clinical diagnostic criteria, ASD is classified into syndromic and non-syndromic types. Syndromic ASD is characterized by core autistic symptoms, accompanied by other phenotypic features or morphological abnormalities, and is frequently observed in clinical practice. Here, it was found that rare syndromes, such as Phelan-McDermid syndrome, Cornelia de Lange syndrome type 1, and cardiofaciocutaneous syndrome type 1, all exhibited features of ASD. Therefore, ASD-related syndromes form a significant component of research into the genetic etiology of ASD. Four cases of tuberous sclerosis complex were identified, caused by mutations in TSC1 and TSC2. One case of Rett syndrome resulted from a MECP2 mutation, and a mutation in NF1 caused one case of neurofibromatosis type 1. All six children were diagnosed with ASD. Tuberous sclerosis, neurofibromatosis, and Rett syndrome all have typical clinical manifestations such as café-au-lait spots, developmental regression and so on. Previous studies have shown that 40%–80% of individuals with genetic syndromes have ASD. Currently known syndromic ASD includes various syndromes caused by mutations in genes such as *MECP2, TSC1, TSC2, NF1*, and *FMR1*, including Rett syndrome and tuberous sclerosis complex (15).

### 4.4 ASD and oncogenes

#### 4.4.1 Syndromic ASD and oncogenes

Research exploring the intersection between ASD and oncogenes has recently emerged as a growing area of interest in genetics. Previous studies have identified TSC1/TSC2 and NF1 as the causative genes for tuberous sclerosis complex and neurofibromatosis type 1, respectively. These results suggest that ASD pathogenesis involves diverse molecular mechanisms that disrupt neural development and function, ultimately contributing to the disorder's complex clinical manifestations (16).

ASD-associated monogenic syndromes are increasingly recognized for their involvement in the onset and progression of other conditions, including cancer and cardiovascular diseases. For example, the PTEN gene negatively regulates the PI3K–AKT–mTOR signaling pathway through its lipid phosphatase activity, controlling cell growth, survival, apoptosis, and metabolism. Mutations in PTEN result in hyperactivation of this pathway, promoting abnormal cell proliferation, impairing neural development and synaptic plasticity, and consequently contributing to the pathogenesis of both ASD and cancer (17).

#### 4.4.2 Extrapolative hypothesis of shared pathogenic genes in guiding drug therapy for ASD

**Figure 2:**
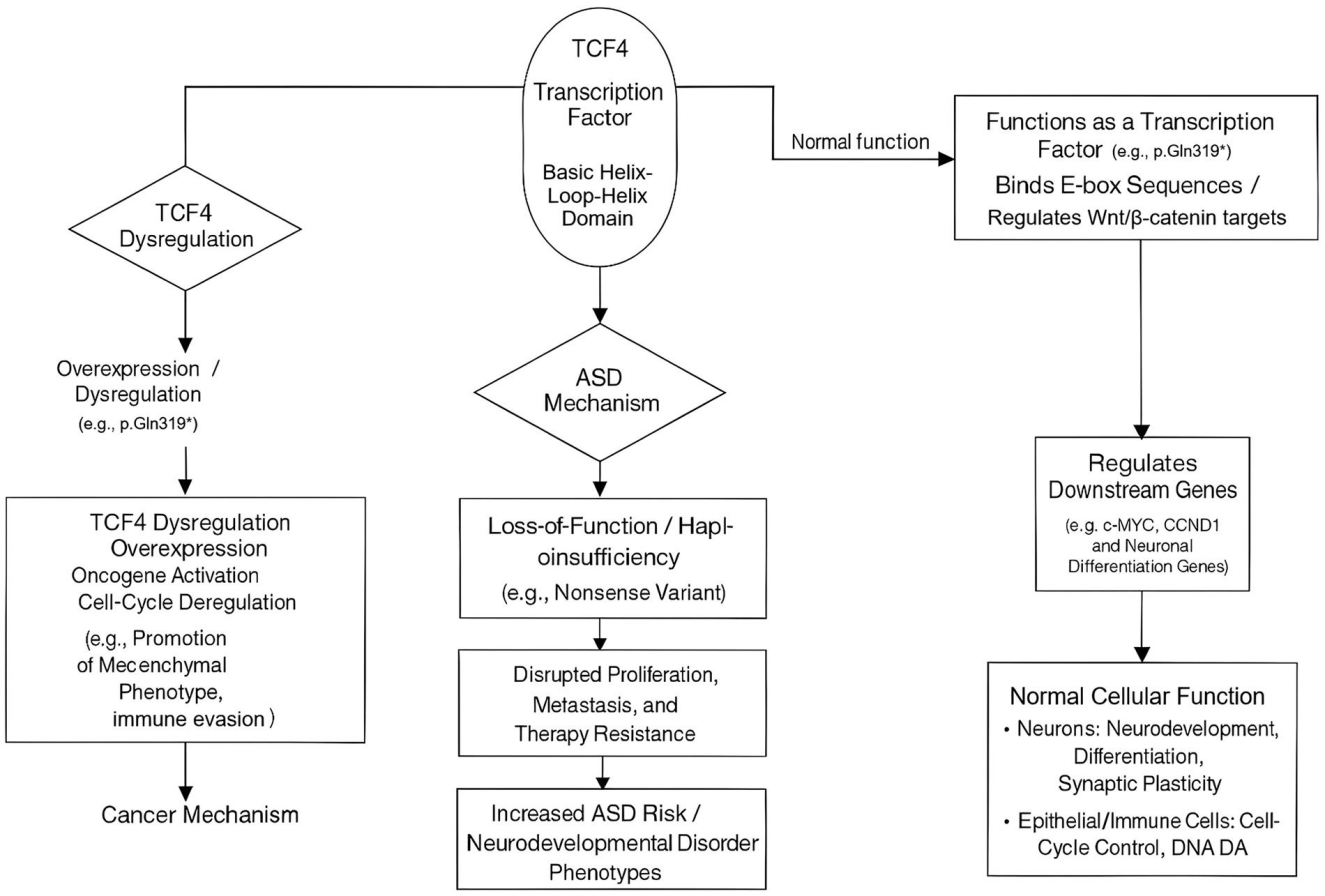
Mechanistic model of *TCF4* function in ASD and cancer. Schematic illustrating normal TCF4 transcriptional activity and its divergent pathological outcomes. Loss-of-function variants such as c.955C > T (p.Gln319*) cause downregulation of neurodevelopmental target genes, leading to ASD-related synaptic dysfunction. At the same time, *TCF4* dysregulation or overexpression promotes oncogenic activation, cell-cycle dysregulation, and immune evasion in cancer.

Mutations in the TCF4 gene are known to cause Pitt–Hopkins syndrome (PTHS), a classical syndrome associated with autism spectrum disorder (ASD). The TCF4 gene, located on chromosome 18q21.2, encodes a basic helix–loop–helix (bHLH) transcription factor that belongs to the E-protein family. It generates multiple transcript variants encoding distinct isoforms. As a transcription factor, TCF4 plays a pivotal role in the Wnt signaling pathway, primarily through its interaction with *β*-catenin, which regulates gene transcription. By activating downstream targets such as c-Myc and Cyclin D1, TCF4 influences key cellular processes including proliferation, differentiation, survival, neuronal migration, and axonal projection (18) (Figure 2).

In 2024, researchers from KU Leuven (University of Leuven), Belgium, and the VIB Center for Cancer Biology published a study in Cell demonstrating the critical role of TCF4-dependent gene regulatory networks in melanoma immunotherapy resistance (19). TCF4 exhibits a degree of genetic overlap between PTHS and melanoma, as the transcription factor encoded by this gene plays essential roles in both neural and melanoma cells. However, their pathological mechanisms diverge significantly: in melanoma, TCF4 contributes to tumorigenesis by regulating the mesenchymal (MES) phenotype and promoting immune evasion, whereas in the central nervous system, TCF4 mutations disrupt normal development and neuronal function, leading to PTHS.

**Figure 3:**
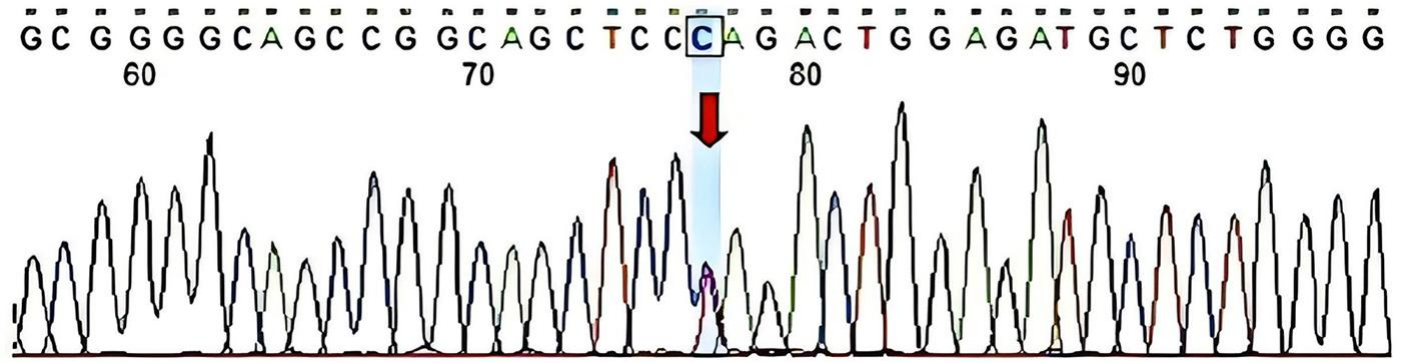
Chromatogram of the TCF4 mutation (heterozygous in the proband).

**Figure 4:**
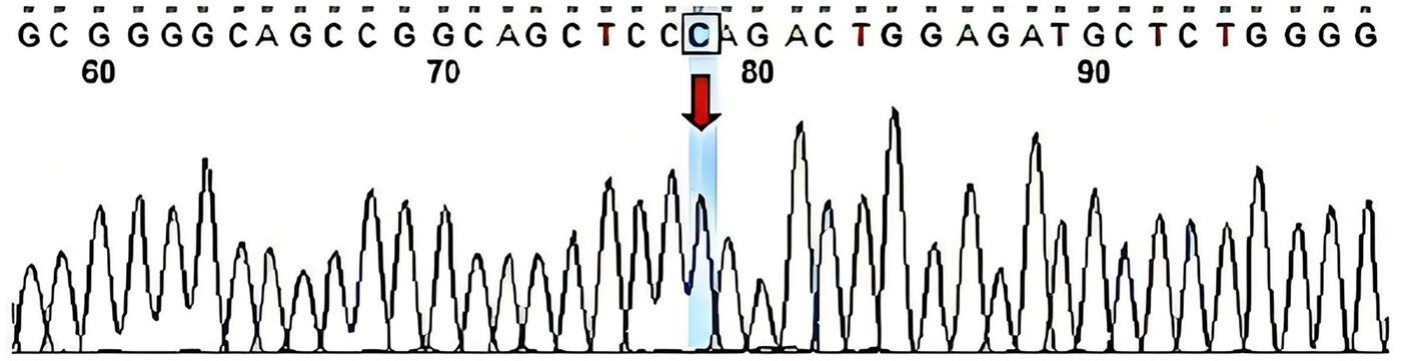
Chromatogram of the TCF4 gene (wild-type in the father).

**Figure 5:**
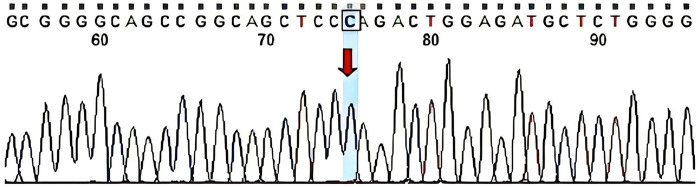
Chromatogram of the TCF4 gene (wild-type in the mother).

Despite these mechanistic distinctions, the shared genetic involvement of TCF4 in both disorders, along with the emerging role of TCF4-targeted therapeutics in melanoma, offers promising insights into potential treatment strategies for PTHS and related ASD conditions. According to the COSMIC database (https://cancer.sanger.ac.uk/cosmic), the most frequently observed TCF4 variants are missense mutations arising from point substitutions, including p.A471 T (c.1411G > A) and p.Q172H (c.516G > T). This study identified a novel TCF4 nonsense variant, c.955C > T (p.Gln319*), which has not been previously reported (Figures 3–5).

### 4.5 Exploration of genotype-phenotype correlations in autism spectrum disorder

Common clinical phenotypes were compared between the positive and negative genetic-test groups, as well as between the CNV and SNV/Indel groups. The results showed that WES analysis significantly improved the rate of positive ASD detection in children with gross motor developmental delays or abnormal EEG results. In clinical practice, active investigation of genetic etiology and pathogenic mechanisms is recommended for children with ASD and concurrent gross motor delay and abnormal EEG findings. Previous studies by Guo et al. (20, 21) have revealed that mutations in certain genes, such as NCKAP1 and CSDE1, are not only associated with autism but are also closely linked to developmental delays (including motor and language delays) and cognitive or learning disabilities. Rapid advancements in genetic testing techniques have led to the identification of an increasing number of pathogenic variants, and WES is now widely used in the diagnosis of NDDs, such as developmental delay and ASD. Large-sample studies on genotype-phenotype correlations in ASD have rarely been reported in China, and this study provides a basis for future research on the genetic etiology of ASD.

### 4.6 Association between ASD severity and genotype

Statistical analysis of disease severity between the positive and negative genetic-test groups revealed significant differences, indicating more severe clinical phenotypes in the positive group. This finding provides a reference for the assessment and clinical management of ASD. Pinto et al. (22) suggested that both CNVs and SNVs play important roles in ASD pathogenesis, with positive CNVs potentially associated with more complex clinical phenotypes, such as those involving intellectual disability and developmental delay. Due to the small number of patients in the CNV group in this study, future research with expanded sample sizes is needed to verify this correlation.

In conclusion, the use of WES significantly enhanced the diagnostic efficacy of genetic analysis of ASD in children with combinations of intellectual disability, gross motor delay, specific facial features, and abnormal EEG results. This study not only expanded the spectrum of genetic mutations and clinical phenotypes associated with ASD but also discovered multiple previously unidentified variants causing ASD, providing a valuable theoretical reference for determining ASD etiology. However, compared with WGS, WES offers somewhat more restricted genomic coverage and exhibits certain inherent constraints in the resolution of CNVs. To potentially improve the diagnostic yield for ASD and uncover a broader spectrum of pathogenic variants, future investigations may benefit from complementary strategies that integrate WGS with orthogonal methods such as array-CGH or CNV-seq (23). Research into genetic etiology is important for prenatal genetic counseling and the development of management strategies for ASD, helping to reduce the rate of congenital disabilities and improve population health. Further advances in genetic sequencing will benefit research into the genetic etiology of ASD.

## Data Availability

The original contributions presented in the study are included in the article/supplementary material. The patient's genetic report are not readily available due to ethical and privacy requirements as they contain private information of the patient and their family members. Further inquiries can be directed to the corresponding author/s.
